# Fine‐Scale Population Structure, High Gene Flow and Local Adaptation in Australasian Snapper (*Chrysophrys auratus*) in New Zealand

**DOI:** 10.1111/eva.70327

**Published:** 2026-10-22

**Authors:** Tom Oosting, Bastiaan Star, Maren Wellenreuther, Peter A. Ritchie

**Affiliations:** ^1^ School of Biological Sciences, Victoria University of Wellington Wellington New Zealand; ^2^ Groningen Institute of Evolutionary Life Sciences University of Groningen Groningen the Netherlands; ^3^ Centre for Ecological and Evolutionary Synthesis (CEES), University of Oslo Oslo Norway; ^4^ The New Zealand Institute for Plant & Food Research Ltd. Nelson New Zealand; ^5^ School of Biological Sciences University of Auckland Auckland New Zealand

**Keywords:** adaptation, gene flow, population genomics, population structure, selection, whole‐genome‐sequencing

## Abstract

Understanding how populations are structured and adapt to their local environment is critical for developing effective management strategies. We used the commercially and culturally valued marine teleost *Chrysophrys auratus* (the Australasian snapper) as an exemplar of a high‐gene‐flow species to study genetic structure, population connectivity, and local adaptation. We performed whole‐genome sequencing of 382 individuals across 13 sample locations, covering the range of the species in New Zealand. We find that snapper consist of two genetic clusters with low levels of genetic differentiation (*F*_ST_ = 0.0031): a northeastern (NE) and a southwestern (SW) cluster, with genetic discontinuities around the Tauroa and Mahia Peninsulas. We further observe widespread admixture and dispersal events, which are indicative of ongoing gene flow, in particular around the areas of the genetic discontinuities near the Tauroa and Mahia Peninsulas. Selection scans identified 25 regions of interest (ROIs) throughout the genome. The haplotype‐based statistic *XP‐nSL* was used to identify which genetic cluster each ROI was most likely under selection, 12 and 13 ROIs for the NE and SW clusters, respectively. The identification of candidate genes with similar molecular functions provided tentative evidence of polygenic adaptation to local environments. Finally, we discuss how monitoring genetic variation contributes to fisheries management by maintaining genetic diversity and resilience in commercial fish. These insights into genetic structure, connectivity, genetic diversity, and local adaptation are critical for protecting species under increased pressure from anthropogenic threats like fishing and climate change, and are thus directly applicable to fisheries management.

## 1 Introduction

Understanding how populations are structured and how they adapt to their environment is a primary goal in population genetics. Population genetic structure in marine fish is often characterized by large effective population sizes and dispersal, resulting in high gene flow and low genetic drift (Nielsen et al. 2009). Low genetic drift typically reduces random allele frequency changes that allow populations to diverge over time, while gene flow homogenizes genetic variation between populations (Slatkin 1985; Star and Spencer 2013). These processes result in low levels of genetic differentiation among neutrally evolving loci. In contrast, low genetic drift also implies that alleles with small selection coefficients have a lower chance of being removed from the population at random, thereby allowing adaptive alleles to spread effectively through a population (Ellegren 2009). While earlier genetic studies using a limited number of loci often had limited power to detect such structure (Cano et al. 2008; Hauser and Carvalho 2008; Ward 2000), advances in sequencing technology have changed how we can study genetic population structure in fish. Coupled with an increasing availability of reference genomes, the acquisition of genome‐wide data has become feasible for many non‐model organisms (Hohenlohe et al. 2020, 2010). The genotyping of thousands to millions of loci provides increased power to detect genetic differences between closely related populations and screen for adaptive loci, including chromosomal rearrangements, gaining novel insights into how wild fish populations are structured and adapt to their environment (Allendorf 2017; Luikart et al. 2018). For example, whole‐genome sequencing has now identified genetic structure and local adaptation in marine fish with low levels of genetic differentiation at various spatial scales, including *Thunnus thynnus* (Andrews et al. 2024; Díaz‐Arce et al. 2024), *Gadus morhua* (Barth et al. 2017; Berg et al. 2016), *Trachurus trachurus* (Fuentes‐Pardo et al. 2023), *Engraulis encrasicolus* (Le Moan et al. 2016), *Engraulis japonicus* (Zhang et al. 2020), *Hippoglossus hippoglossus* (Kess et al. 2021), and *Clupea harengus* (Han et al. 2020).

The Australasian snapper or tāmure ( *Chrysophrys auratus* ) is a commercially important marine fish in New Zealand and holds high cultural value as a *taonga* species for the indigenous Māori. Snapper primarily occurs in inshore waters around the North Island and the top of the South Island and are generally found in coastal waters to a 50 m depth (Kendrick and Francis 2002), but can be found to a 200 m depth (Crossland 1981). Since the onset of industrialized fishing, snapper have experienced significant population declines but have shown a recovering trend (Ministry for Primary Industries 2013). Snapper are serial broadcast spawners, releasing millions of eggs during large spawning aggregations, for which they can travel up to hundreds of kilometers (Parsons et al. 2014). During their pelagic stages, eggs and larvae can travel long distances before settling in sheltered estuarine habitats (Scott et al. 1993; Zeldis 1993). These traits are important factors in the dispersal and spatial structuring of snapper. Based on differences in year‐class strength, age structure, and growth rates, fisheries management recognizes eight demographically distinct stocks, which are managed across four fishery areas (Figure 1, left inset and Table S1). Three stocks (Northland, Hauraki Gulf, and Bay of Plenty) are recognized in SNA1 (Walsh et al. 2011), while two stocks (northern and southern) are recognized in SNA2, located on either side of the Mahia Peninsula (Walsh et al. 2012). Snapper north of the Mahia Peninsula showed similar year‐class strength to the Bay of Plenty stock in SNA1 (Walsh et al. 2012), suggesting some degree of demographic connectivity. The presence of two separate stocks has been hypothesized for SNA7 (Marlborough Sounds and Tasman Bay) (Fisheries New Zealand 2018), and SNA8 is thought to consist of a single stock (West Coast) (Walsh et al. 2017, 2006). Despite these insights, there have been no fine‐scale analyses of their genetic structure, genetic diversity, and putative adaptation to local environments using whole‐genome sequencing approaches. This knowledge will be critical for determining how environmental pressures may impact genetic diversity and will help assess the potential evolutionary resilience of populations to environmental changes, thereby informing sustainable fisheries management (Heuertz et al. 2023).

**FIGURE 1:**
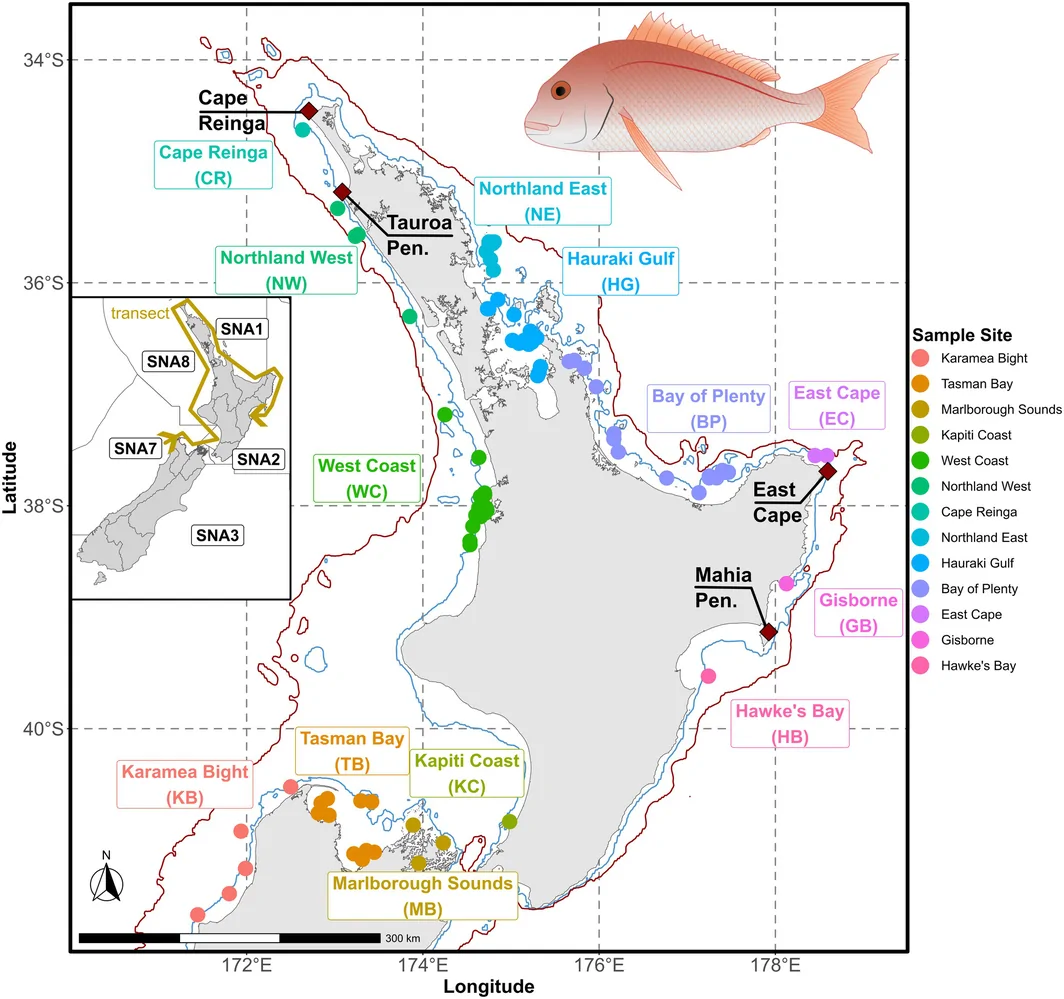
Map showing sample distribution in New Zealand. Colors indicate individual sample locations. Approximate sample locations were used for Hawke's Bay and Gisborne as exact fishing locations were not available for these sites. Blue contour lines show 50 m bathymetry line where snapper primarily occur, and the red lines indicate the 200 m bathymetry line, indicating the maximum depth where snapper is found. Left inset shows the transect along which results will be shown (Table S3). Top right shows an illustration of snapper (Courtesy of Plant and Food Research).

So far, genetic studies using allozyme and microsatellite data have shown the presence of two genetic clusters (Bernal‐Ramírez et al. 2003; Smith et al. 1978). Both studies observed significant genetic differentiation between sample locations on either side of Northland and the Mahia Peninsula. Intriguingly, both studies identified that individuals from Hawke's Bay were genetically closest to those from the West Coast (Bernal‐Ramírez et al. 2003; Smith et al. 1978), despite these sample locations being situated on opposite sides of the North Island (Figure 1). Bernal‐Ramírez et al. (2003) also observed a barrier to gene flow using microsatellites around the Cook Strait, which separates Tasman Bay from the North Island (Figure 1), whereas such separation was not detected using allozymes (Smith et al. 1978). Bernal‐Ramírez et al. (2003) hypothesized that microsatellite loci could have a higher sensitivity to detect recent population differentiation facilitated by genetic drift. Tasman Bay had experienced a severe population decline, reducing it to 7% of its original biomass (Annala et al. 2000). This population decline led to significant genetic drift and a loss of genetic diversity in microsatellite loci (Hauser et al. 2002). In addition to genetic differentiation among neutral loci, Smith et al. (1978) also showed that snapper from Hawke's Bay were genetically more similar to those from the West Coast than those from the East Cape for an allozyme locus linked to an esterase gene. This was striking because Hawke's Bay and the East Cape are in much closer proximity compared to the West Coast, spurring the hypothesis that this locus was under selection since allele frequencies for this locus significantly correlated with changes in ocean temperature over time (Smith 1979). These studies show that both neutral and putatively adaptive loci exhibit similar genetic structure, suggesting that potentially barriers to dispersal and local adaptation may contribute to the genetic differentiation observed in snapper. No apparent genetic structure could be observed using mitochondrial markers (Ashton 2013; Bernal‐Ramírez et al. 2003; Smith et al. 1978), a finding consistent with recent mitogenomic work, which showed that patterns of mitochondrial variation reflect long‐term and broad‐scale evolutionary processes (Oosting et al. 2023). The discrepancy between genetic structure and stock structure recognized by fisheries suggests that gene flow is high between demographically independent stocks, making them genetically indistinguishable from each other. However, it is possible that the relatively limited number of loci used in previous studies had insufficient power to detect even further fine‐scale genetic structure (Cano et al. 2008; Morin et al. 2010).

Given the unresolved genetic structure of snapper and the apparent disparity of two clusters with existing management recognizing eight demographically distinct stocks, there is a need to better understand how Australasian snapper in New Zealand are genetically structured. Moreover, little is known about how wild snapper respond to their environment and whether certain stocks harbor adaptive variation that is specific to them. Such observations would have serious implications for fisheries management, as losing these stocks would mean losing adaptive genetic variation specific to that area. This is particularly relevant today because New Zealand's marine environment is under increasing threat from climate change and is experiencing changes in acidity, primary production, ocean circulation, and temperature, with some areas experiencing 34% higher increases in water temperature compared to the global average (Ministry for the Environment and Stats NZ 2025; Pinkerton et al. 2024). To address these questions, we analyzed 382 whole‐genome sequences from snapper samples collected at 13 locations across most of New Zealand's geographic range, covering all stocks identified by fisheries management. First, we assessed genetic structure and population connectivity to resolve fine‐scale population structure. Second, we tested for evidence of local adaptation by detecting genomic regions under putative selection in the identified genetic clusters using selection scans. Finally, we assess levels of genetic diversity and discuss why monitoring such variation is crucial for maintaining population resilience. Our study provides an example of how whole genome data provides fundamental insights into the genetic structure, connectivity, genetic diversity, and local adaptation of a high‐gene‐flow species, with implications for conservation management under fishing and climate change impacts.

## 2 Materials and Methods

### 2.1 Sampling and DNA Extraction

Fin clips were collected for 382 individuals between 2017 and 2022 from 13 sample locations: Karamea Bight (*N* = 19), Tasman Bay (*N* = 33), Marlborough Sounds (*N* = 34), Kapiti Coast (*N* = 17), West Coast (*N* = 37), Northland West (*N* = 19), Cape Reinga (*N* = 10), Northland East (*N* = 18), Hauraki Gulf (*N* = 71), Bay of Plenty (*N* = 69), East Cape (*N* = 20), Gisborne (*N* = 15), Hawke's Bay (*N* = 20) (Figure 1, Tables S1, S2). Samples were obtained from commercial and recreational fishing sources, and no fish were killed specifically for this study. Samples were preserved in DESS (20% DMSO, 0.5 M EDTA pH 8, and saturated NaCl) and stored at −20°C (Oosting et al. 2020). DNA was isolated using a high‐salt extraction protocol adapted from Aljanabi and Martinez (1997). Purified DNA was stored in 10 mM Tris–HCl pH 8 and 0.1 mM EDTA at −20°C. DNA quantity was determined using the Qubit broad‐range kit (> 20 ng/μL) (Invitrogen), following the manufacturer's instructions. Sample quality was assessed using an Implen NP80 spectrophotometer (260/280 < 2.0 and/or 260/230 > 2.0) and gel electrophoresis, selecting samples with high‐molecular‐weight DNA (> 20kbp fragments).

### 2.2 Whole‐Genome‐Sequencing

Library preparation and whole‐genome sequencing of 370 samples were performed by the Australian Genome Research Facility (AGRF). Library preparation was performed using the Nextera Flex protocol with unique dual indexing. Sequencing was performed using the Illumina NovaSeq 6000 S4, generating 150 bp paired‐end reads with an average sequencing depth of ~11× per individual. Reads from 12 additional individuals from a pilot study were also included (unpublished data). Samples were sequenced using the Illumina HiSeq 4000, generating 100 bp paired‐end reads with an average sequencing depth of ~30× per individual. The reads for these 12 individuals were generated in two separate lanes.

### 2.3 Read Alignment

Read quality was assessed using FastQC v0.11.7 (Andrews 2010) and compiled with MultiQC v1.7 (Ewels et al. 2016). Reads were aligned to both the nuclear and mitochondrial snapper reference genomes using PALEOMIX v1.2.13.3, using the BAM pipeline (Ashton 2013; Catanach et al. 2019; Schubert et al. 2014). The nuclear reference consists of 24 pseudo‐chromosomes, reflecting the known number of chromosomes in this species (Catanach et al. 2019). First, Adapter Removal v2.2.3 was used for trimming Illumina forward and reverse adapters, allowing a 33% mismatch rate (−‐mm 3) and a minimum read length of 25 bp. N's and low‐quality bases were trimmed off the 5′ and 3′ ends. The Burrows‐Wheeler Aligner (BWA) v0.7.15 (bwa‐mem algorithm) was used to align reads (Li and Durbin 2009), using a minimum mapping quality of 25. MarkDuplicates and Picard tools v2.18.20 was used to remove PCR duplicates (http://broadinstitute.github.io/picard/), and local realignment was performed using GATK v4.0.8.1 (Van der Auwera and O'Connor 2020). Soft‐clipped reads were removed using SAMtools v1.9 (Danecek et al. 2021), and BAM files were normalized for depth of coverage by randomly subsampling individuals with significantly more data down to the mean depth using SAMtools view (Danecek et al. 2021).

### 2.4 Genotyping and SNP Filtering

Genotypes were identified using BCFtools (Li 2011). First, all‐sites VCF files were generated for each linkage group (pseudo‐chromosomes) using BCFtools mpileup and call (−m) (Li and Durbin 2009). Genotypes with a sequencing depth below three (DP < 3) and a genotype quality below 20 (GQ < 20) were set to missing using BCFtools filter. Subsequently, BCFtools view was used to remove indels and multi‐allelic sites (STRLEN(REF)! = 1 | STRLEN(ALT) > = 2), and biallelic sites were filtered for quality below 600 and mean coverage between 8 and 25× (QUAL < 600 | AVG(FMT/DP) < 8 | AVG(FMT/DP) > 25). AllSites VCF files were tab indexed using HTSlib v1.9. Annotations were added using BCFtools +fill‐tags, and VCF files from each linkage group were merged using BCFtools concat.

Second, we extracted all high‐quality biallelic SNPs (‐‐max‐alleles 2 ‐‐min‐alleles 2) and removed low‐quality SNPs (‐‐max‐missing 0.95 ‐‐maf 0.01) using VCFtools v0.1.16 (Danecek et al. 2011). A custom script adapted from Pinsky et al. (2021) was used to remove heterozygous SNPs that were subjected to allelic imbalance. In short, the script performs a two‐sided binomial test to assess whether the reference and alternative alleles occur in equal proportions at heterozygous sites. SNPs that significantly deviated from equal proportions were excluded from the dataset. The retained high‐quality SNPs were used to identify outlier and neutral loci.

Outliers were identified using OutFlank (Whitlock and Lotterhos 2015) and pcadapt (Luu et al. 2017). OutFlank was run using a minimum heterozygosity of 0.1 (Hmin 0.1), and LeftTrimFraction and RightTrimFraction fraction of 0.05 according to the author's recommendations (Whitlock and Lotterhos 2015). We used sample locations to partition samples into groups. Loci with a false discovery threshold below 1% (*q* < 0.01) were considered outliers. For pcadapt, we first ran the program with ten principal components (*K* = 10) and used a scree plot to determine the number of informative principal components using Cattell's rule (*K* = 1) (Figure S1). Outliers were identified for *K* = 1 informative principal component using a minimum allele frequency of 0.05 (min.maf 0.05). Again, loci with a false discovery threshold below 1% (*q* < 0.01) were considered outliers. Overlapping outliers between OutFlank and pcadapt were retained and subsequently thinned using a non‐overlapping sliding window of 50,000 bp to obtain independently segregating sites. For each sliding window, the locus with the highest *F*_ST_ value was retained.

A neutral SNP dataset was obtained by first removing all outlier loci (OutFlank and pcadapt). Second, retained SNPs were filtered for deviations from Hardy–Weinberg equilibrium (‐‐hwe 0.05) and a minimum allele frequency (‐‐maf 0.05), and were thinned (‐‐thin 5000) using VCFtools v0.1.16 (Danecek et al. 2011). The appropriate window size for thinning was determined by estimating the average linkage decay (Figure S2). Finally, linked loci were removed using Plink (‐‐indep‐pairwise 50 5 0.2) (Purcell et al. 2007). After SNP filtering, we obtained four datasets, one containing all loci including uninformative sites (i.e., All Sites), and three biallelic datasets (i.e., high‐quality, outlier, and neutral).

### 2.5 Genetic Structure and Demographic Connectivity

Principal component analyses (PCAs) were performed on biallelic datasets using SNPRelate (Zheng et al. 2012) and visualized using a custom script obtained from github.com/therkildsen‐lab/genomic‐data‐analysis. Global average and pairwise levels of population differentiation were estimated by calculating *F*_ST_ from all SNPs using the snpgdsFST function in SNPRelate (Zheng et al. 2012). *F*_ST_ estimates were weighted by sample size across all loci following (Weir and Cockerham 1984). Pairwise‐*F*_ST_ estimates between sample locations were visualized using the R‐package pheatmap (Kolde and Kolde 2015). Pheatmap clustered sample locations and generated a dendrogram showing similarities between sample locations. A Mantel test was used to test for isolation by distance (IBD) between sample locations using the R‐package ade4 (Thioulouse et al. 2018), using linearized genetic distance (*F*_ST_ /1 – *F*_ST_) and the log_10_ of geographic distance in km. Geographic distances were estimated by calculating the least‐cost distance using the R‐package gdistance (Table S3) (van Etten 2017).

The maximum likelihood approach implemented in ADMIXTURE was used to estimate individual ancestry levels and the most likely number of clusters (Alexander et al. 2009). ADMIXTURE was run using neutral loci and tested for K clusters between 1 and 4 by performing 10,000 iterations. The number of K clusters that best explained the structure in the dataset was evaluated using cross‐validation by selecting the K clusters with the lowest error. Individuals were assigned to a genetic cluster based on their highest level of ancestry to one of K clusters. To identify where spatial discontinuities in the levels of ancestry to a specific population were strongest, we plotted the mean level of ancestry to a specific cluster along a transect (see the panel in Figure 1 for the transect). Sample locations were grouped based on their highest level of mean ancestry to one of the K clusters.

Relative migration rates were estimated using divMigrate included in the Rpackage diveRsity to estimate relative rates of migration (Keenan et al. 2013). We used *G*_ST_ as a measure of genetic differentiation, and 10,000 iterations were performed to test for asymmetrical migration. Relative migration rates between the identified clusters were estimated using both neutral and independent outlier loci.

### 2.6 Genetic Diversity

Genotype statistics for each sample were calculated using BCFtools stats, and levels of heterozygosity were estimated for all four datasets. An ANOVA was performed on all biallelic datasets to test for significant differences in heterozygosity among sample locations and genetic clusters using R (R Core Team 2013). A hierarchical analysis of molecular variance (AMOVA) was performed using the R‐package poppr (Kamvar et al. 2014). A hierarchical model was applied to compare the variation among sample locations and to identify genetic clusters. Significance levels were tested using randtest from the R‐package adegenet, using 999 permutations.

### 2.7 Selection Scans

To test for evidence of selection, we performed a selection scan with pixy v2.0.0.beta14 (Korunes and Samuk 2021). Pixy includes both variant and invariant sites to accurately estimate summary statistics by adjusting for missing data. Using the Allsites dataset, we estimated relative population divergence (*F*_ST_) over 10,000 bp nonoverlapping sliding windows. In addition, *XP‐nSL* was estimated using selscan (v3.0.0) using the ‐‐unphased flag (Szpiech 2024). *XP‐nSL* is a haplotype‐based statistic that can detect selection by estimating the mean number of sites in a region that are identical by state across all pairs of haplotypes, contrasting haplotype sets in two different populations. *XP‐nSL* specifically tests for differences in the length of high‐frequency haplotypes and diversity between populations, without requiring a linkage map or the ancestral state of each SNP (Szpiech et al. 2021), making *XP‐nSL* an ideal summary statistic for studies with limited access to such genomic resources. Moreover, *XP‐nSL* has been shown to have similar power to detect selection using unphased datasets as with phased data (Szpiech 2024). One drawback is that *XP‐nSL* gives a ratio of the selection strength between our two genetic clusters rather than an absolute measure of selection. However, we were primarily interested in identifying which regions are most likely involved in local adaptation within each genetic cluster, making *XP‐nSL* an ideal summary statistic given our current data. Output was normalized using selscan norm and summarized over 10 kbp nonoverlapping sliding windows.

Genomic regions under putative selection were identified by selecting 10 kbp sliding windows that were in the top 1% of both *F*_ST_ and *XP‐nSL* results (windows of interest, or WOIs). Identifying genomic regions that share these two signals is critical, as they can also experience substantial increases in genetic divergence in the absence of selection via genetic drift (Booker et al. 2021). Selected windows were subsequently grouped into regions of interest (ROIs) based on their distance (with a max 100 kbp distance). To minimize false positives, ROIs had to contain at least two WOIs. Regions were classified as being under selection in a specific population based on the *XP‐nSL* value. Results were visualized using Manhattan plots generated in R using a custom script.

Finally, we performed a gene set enrichment analysis using GOfuncR to test for selection on specific traits (Grote 2025). Genes within the regions of interest were extracted from the snapper annotation produced by Catanach et al. (2019), containing 22,424 annotated genes. Using the go_enrich function, we tested the overrepresentation of specific GO terms using the hypergeometric approach, which compares the presence of specific GO terms against a background gene set (i.e., the full annotation). To identify more relationships among genes, we manually curated gene functions and looked for overarching patterns.

## 3 Results

### 3.1 Sequencing and SNP Filtering

In total, 14.6 billion reads were generated over 382 individuals, with an average of 38.2 million reads per individual. Sequencing depth ranged between 7.17× and 60.1×, with an average of 10.8× per individual. After read alignment, BAM files of 48 individuals with significantly more reads compared to the average were randomly subsampled to remove bias between diversity estimates (Figure S3). BCFtools genotyped 11.4 million SNPs, and 4,185,623 high‐quality SNPs were retained after removing indels and low‐quality SNPs. High‐quality SNPs showed a missing rate of 0.8%. OutFlank identified 3843 outlier loci with a false discovery rate of 1% (*q* = 0.01). Pcadapt identified 6008 outlier loci with a false discovery rate of 1% (*q* = 0.01). Combined, outlier analyses identified 6448 unique outliers, of which 3403 overlapped. After removing presumed linked loci, the final outlier dataset consisted of 278 independently segregating loci after thinning. The neutral dataset consisted of 69,700 independently segregating loci after removing all 6448 outlier loci, non‐independently segregating sites (thinning & loci in linkage disequilibrium), and sites deviating from Hardy–Weinberg. The percentage of missing data among neutral SNPs was 0.9%.

### 3.2 Demographic Analyses

Principal Component Analysis showed the presence of two genetic clusters (Figure 2A, Figure S4): one cluster occurring along the northeast of the North Island (the NE cluster) and one occurring along the southwest of the north Island and the top of the South Island (the SW cluster). Only the first axis of the PCA was informative for inferring genetic structure and explained a small proportion of the total amount of variation present in the neutral data (0.59%) (Figure 2A). Three sample locations consisted of individuals from both genetic clusters (Cape Reinga, Gisborne, and Hawke's Bay), showing dispersal between genetic clusters.

**FIGURE 2:**
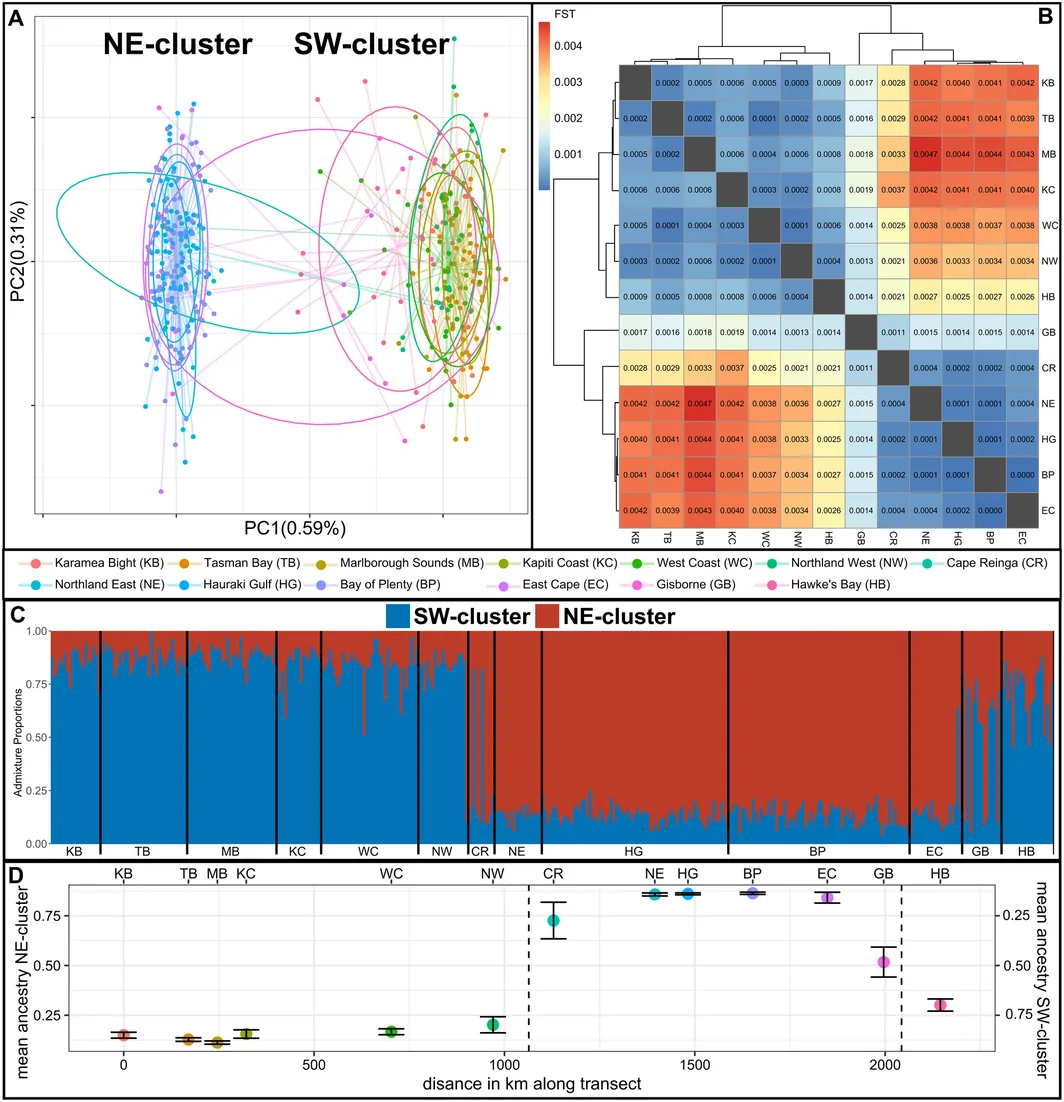
Demographic analyses using 67,900 neutral loci of snapper in New Zealand. (A) PCA based on a genetic relationship matrix using neutral loci showing genetic structure. Individuals were grouped by sample location. Ellipses indicate the sample spread for each location, with lines connecting individual samples to the centre of their respective ellipse. Similar PCAs based on high‐quality and outlier loci are shown in Figure S5 (B) Heatmap showing pairwise weighted FST estimates for sampling locations. Estimates for each pair‐wise comparison are depicted inside each square. Colors show the degree of differentiation among pairwise comparisons. The dendrogram indicates which sample locations are genetically most similar based on pairwise FST estimates. (C) Admixture plot for *K* = 2. Admixture proportions correspond with PCA results. Blue shows ancestry to the SW cluster, red shows ancestry to the NE cluster. Sample locations are ordered along the transect shown in Figure 1 and separated by black vertical lines (D) Mean levels of ancestry to NE cluster (left *y*‐axis) and SW cluster (right *y*‐axis), plotted along the transect shown in Figure 1. Error bars show the standard deviation, dotted vertical lines show the locations of the proposed genetic discontinuities (left: Tauroa Peninsula, right: Mahia Peninsula). Sample location acronyms are shown above the plot panel.

Pairwise‐*F*_ST_ estimates also reflected the observed genetic structure (Figure 2B, Table S4). The dendrogram grouped sample locations into two distinct clusters. The NE cluster consists of Cape Reinga, Northland East, the Hauraki Gulf, the Bay of Plenty, East Cape, and Gisborne. The SW cluster consists of the Karamea Bight, Tasman Bay, the Marlborough Sounds, the Kapiti Coast, the West Coast, Northland West, and Hawke's Bay. Global average genetic differentiation among sample locations using neutral loci was *F*_ST_ = 0.0022, while genetic differentiation between the NE cluster and SW cluster was *F*_ST_ = 0.0031. Pairwise‐*F*_ST_ estimates between sample locations ranged between 0.00003 and 0.0047, with comparisons within clusters ranging from 0.00003 to 0.0015, and between clusters ranging from 0.0013 to 0.0047. Isolation by distance was detected across the range of snapper using a Mantel test (*p* = 0.011); however, sample locations within the SW and NE cluster did not show a similar trend (*p* = 0.344 and 0.698, respectively) (Figure S5).

ADMIXTURE results showed the highest likelihood for *K* = 2 (Figure S6), also supporting the presence of two genetic clusters. Ancestry levels showed that practically all individuals had a mixed ancestry (Figure 2C). Notably, individuals that showed intermediate levels of ancestry to both genetic clusters were present only south of the genetic discontinuities. Mean levels of ancestry for each sample location were used to group them into genetic clusters (Figure 2D). The strongest spatial discontinuities in the levels of ancestry were identified between Northland West and Cape Reinga and Gisborne and Hawke's Bay (Figure 2D), with genetic discontinuities identified around Tauroa Peninsula and Mahia Peninsula, respectively (Figure 1). Sample locations Cape Reinga, Gisborne and Hawke's Bay showed intermediate levels of mean ancestry (maximum ancestry < 0.75), suggesting that the mixing of individuals from both genetic clusters occurs at these locations.

Relative migration between genetic clusters was symmetrical among neutral loci (Table S5). In contrast, relative migration among outlier loci was 5% higher from the NE cluster to the SW cluster (Table S5), suggesting asymmetrical migration.

### 3.3 Genetic Diversity

Genetic diversity varied among sample locations and the identified genetic clusters. An analysis of molecular variance (AMOVA) showed that the majority of variation was observed within individuals (Table 1): 99% and 84% for neutral and outlier loci, respectively. Only 0.28% of neutral variation was observed between genetic clusters, while variation between genetic clusters in outlier loci was 12.27%. Both neutral and outlier loci showed little variation among sample locations within genetic clusters and among samples within sample locations (< 0.8%) (Table 1). Levels of heterozygosity varied between datasets (Table S1, Figure S7). Mean heterozygosity was lowest in high‐quality loci (0.215), with neutral and outlier loci having a mean heterozygosity of 0.301 and 0.372, respectively. The observed difference between the high‐quality dataset and the neutral and outlier datasets is caused by different minimum allele frequency thresholds, 0.01 and 0.05, respectively. Heterozygosity also varied among sample locations, ranging from 0.213 to 0.218, 0.298 to 0.304, and 0.359 to 0.393 for high‐quality, neutral, and outlier loci, respectively. Significant differences were observed between 13 and 11 population comparisons for high‐quality and neutral loci, respectively, but none were observed between sample locations for outlier loci (Figure S7). Among high‐quality and neutral loci, Northland East, the Hauraki Gulf, and the East Cape showed higher levels of heterozygosity compared to the Karamea Bight, Tasman Bay, the Marlborough Sounds, and Gisborne (Figure S7). Heterozygosity was significantly different between genetic clusters (Figure 3), with heterozygosity among high‐quality and neutral loci being higher in the East cluster, while heterozygosity among outlier loci was significantly higher in the West cluster. The mean level of heterozygosity among all samples and all loci (including invariant sites) did not vary significantly among sample locations and between genetic clusters (mean *H* = 0.00328).

**TABLE 1:** AMOVA performed using neutral and outlier loci.

	d.f.	Sum sq	Mean sq	σ	%	ф	p
**Neutral loci**							
Between GCs	1	23866	23866	34.21	0.32	0.00322	0.001
Among SITES within GCs	11	118599	10781	2.67	0.02	0.00025	0.001
Among individuals within SITES	369	3924435	10635	69.51	0.65	0.00657	0.225
Within individuals	382	4009587	10496	10496.3	98.99		
Total	763	8076488	10585	10602.7	100		
**Outlier loci**							
Between GCs	1	3257	3257	8.29	13.43	0.134	0.001
Among SITES within GCs	11	950	86	0.59	0.97	0.011	0.001
Among individuals within SITES	369	19802	53	0.8	1.3	0.015	0.074
Within individuals	382	19886	52	52.05	84.29		
Total	763	43897	57	61.75	100		

Notes: GCs = Genetic clusters, SITES = sample locations, d.f. = degrees of freedom, Sum sq = sum of squares, Mean sq = mean of squares σ = variance, % = percentage of variance explained, ф = phi‐statistics, *p* = p‐value.

**FIGURE 3:**
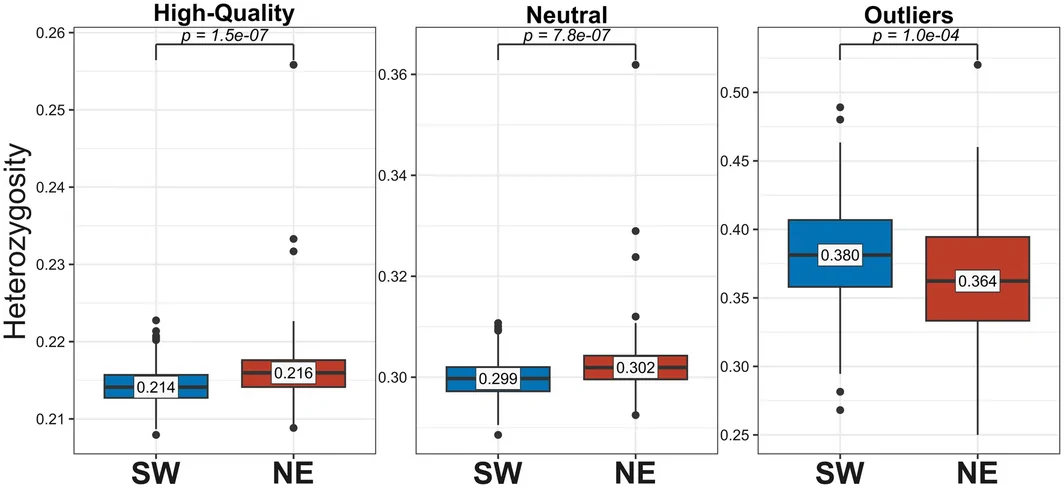
Box plots showing levels of heterozygosity among individuals between the NE cluster and SW cluster for 4,185,623 high‐quality loci, 67,900 neutral loci, and 278 independent outlier loci. Mean heterozygosity levels are shown in the middle of each boxplot. Levels of significance between genetic clusters for each SNP dataset are shown at the top of each panel.

### 3.4 Identification of Local Adaptation and Candidate Loci

Genomic regions with elevated levels of genetic divergence and signatures of local adaptation were detected across the entire genome (Figure 4). In total, 25 genomic regions of interest (ROIs) exhibited significant levels of genetic differentiation, of which 12 regions showed evidence of being under selection in the SW cluster and 13 in the SW cluster (Table S6). The sizes of these ROIs ranged from 20 to 490 kbp. Of these 25 ROIs, 19 contained coding regions for 78 genes, of which 48 were potentially under selection in the NE cluster and 31 in the SW cluster (one gene was identified for both clusters). Gene set enrichment analysis did not show any overrepresentation of GO terms in either genetic cluster, suggesting that the candidate genes are not involved in similar functional groups. However, manual curation provided tentative evidence that the candidate genes are involved in similar functional groups (Table S7). Most candidate genes grouped were involved in broad functional pathways, including hormone or neuronal signaling, and transcription or chromatin regulation. However, four genes distributed across two ROIs on linkage groups 6 and 24 in the SW cluster suggested selection on glycogenesis (angptl3, leprot, pgm1, cds2, prkag2, gyg1, and mdh1b) (Table S7).

**FIGURE 4:**
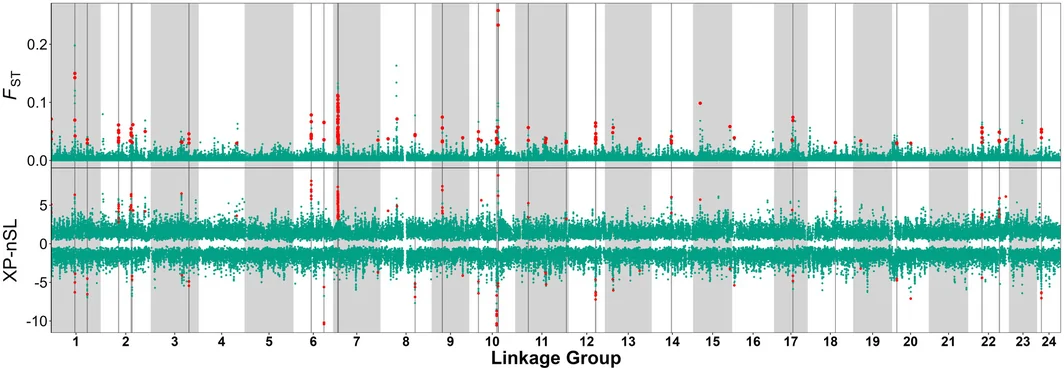
Manhattan plot showing *F*_ST_ and *XP‐nSL* for 10,000 base pair non‐overlapping windows. Significantly differentiated sliding windows are shown in red, and 25 regions of interest are indicated by vertical black lines. Linkage groups (pseudochromosomes) are shown on the *x*‐axis. Note that for *XP‐nSL* we show the most divergent estimate for that window to best capture selection occurring in both directions, and thus intermediate values near 0 are absent.

## 4 Discussion

### 4.1 Genetic Structure of New Zealand Snapper

Here we report genome‐wide data showing that snapper in New Zealand consist of two main genetic clusters (i.e., the NE cluster and the SW cluster), with genetic discontinuities around the Tauroa and Mahia Peninsula (Figure 5). This separation was supported by a range of different datasets and analyses. First, PCA for both the neutral (69,700 SNPs) and outlier (278 SNPs) datasets clearly separated the majority of individuals into the two distinct groups (Figure 2A, Figure S4). Using neutral loci, the first two principal components (PC1 and PC2) explained a small proportion (0.59% and 0.31%) of the total variation—likely due to high levels of individual variation among specimens—whereas outlier SNPs explained significantly more variation (16.06%) along PC1. Second, mean ancestry proportions from ADMIXTURE supported the same structure, with strong changes in mean ancestry proportions around the Tauroa and Mahia Peninsula (Figure 2D). These observations provide genome‐wide support for the observations of previous studies (Bernal‐Ramírez et al. 2003; Smith et al. 1978). Our study also shows that geographically isolated sample locations, the West Coast and Hawke's Bay, belong to the same genetic cluster (Figure 2B), supporting the hypothesis that these stocks are connected via the D'Urville current that runs through the Cook Strait (Bernal‐Ramírez et al. 2003). In contrast to Bernal‐Ramírez et al. (2003), we did not find evidence of the presence of a separate genetic stock in Tasman Bay (Figure 2). While it is possible that increased genetic drift caused by a drastic population decline in Tasman Bay resulted in genetic differentiation in highly polymorphic microsatellite loci, our genomic data indicate there is no evidence to suggest that Tasman Bay is a genetically divergent population.

Low but significant genetic differentiation between genetic clusters is biologically relevant for population management (Knutsen et al. 2011), and our observations are typical for a widespread marine species characterized by large effective population sizes (Bertram et al. 2024). The level of genetic differentiation (*F*_ST_) observed between genetic clusters was 0.0031, while global‐*F*_ST_ among sample locations was 0.0022. In Australia, snapper populations along the southwest and southeast coasts showed comparable levels of genetic differentiation (global‐*F*_ST_ = 0.0041 and 0.0116, respectively) (Bertram et al. 2023, 2022). Others include Atlantic cod (*Gadus morhua, F*_ST_ = 0.0031), Atlantic herring ( *Clupea harengus* , *F*_ST_ = 0.013–0.061), Atlantic horse mackerel ( *Trachurus trachurus* , *F*_ST_ = 0.002–0.012), and bluefin tuna ( *Thunnus thynnus* , *F*_ST_ = 0.0004–0.0018) (Andrews et al. 2024; Barth et al. 2017; Fuentes‐Pardo et al. 2023; Han et al. 2020).

We further observe a lack of population substructure within each genetic cluster using both neutral and outlier loci (Figure 2, Figure S4), while four independent stocks have been identified by fisheries based on growth data and year‐class strength (Walsh et al. 2017, 2006, 2011, 2012). The lack of substructure suggests that the eight demographically independent stocks are unlikely to harbor adaptive genetic variation specific to that stock. This is positive from a management perspective, as it reduces the risk of losing unique genetic variation due to population declines. Levels of genetic differentiation between sample locations (which largely reflect stock structure) within genetic clusters were very low, ranging from 0.0000 to 0.0008 (Figure 2B; excluding locations where mixing was observed, see below), which is an order of magnitude lower than that observed between the two genetic clusters. Moreover, while significant IBD suggested that genetic similarity is reduced over large spatial scales (Figure S5), this pattern was entirely driven by the genetic discontinuities and implies that the genetic variation within genetic clusters is largely homogeneous. This highlights the apparent mismatch between the demographically independent stocks based on demographic traits and the observed genetic structure. Theoretical models explain this mismatch because only a small number of migrants is required to homogenize gene pools, while substantially more individuals are needed to homogenize population demography (Lowe and Allendorf 2010; Waples and Gaggiotti 2006). This disparity between demographic and genetic structure has important implications for fisheries management. For example, overfishing of a specific demographically independent stock does not necessarily imply the loss of genetic diversity, which can be replenished over time once overfishing has stopped. The genetic structure observed here implies that fisheries management of Australasian snapper in New Zealand can focus on the maintenance of genetic variation within two large gene pools.

Interestingly, the observed genetic discontinuities around the Tauroa and Mahia Peninsula have also been reported in other marine species. These include tarakihi ( *Cheilodactylus macropterus* ), barracouta (*Thyrisites atun*), jack mackerel ( *Trachurus declivis* ) (Gauldie and Johnston 1980; Papa et al. 2020), the waratah anemone ( *Actinia tenebrosa* ) (Veale and Lavery 2012), and the New Zealand Estuarine Clam (*Austrovenus stutchburyi*) (Ross et al. 2012). Moreover, the area between Cape Reinga and the Tauroa Peninsula (90‐mile‐beach) has been identified as a prominent genetic break for a further 21 marine species (Arranz et al. 2021), and was proposed as a boundary between bioregions based on macroalgae and benthic invertebrate communities (Shears et al. 2008). It is likely that ocean currents play a significant role in dispersal around these genetic discontinuities. Such currents particularly impact species during early pelagic life stages, when larvae drift along with the currents until settling in shallow coastal and estuarine‐like habitats (Parsons et al. 2014). It is possible that strong ocean currents also influence dispersal during the adult life stage. At Cape Reinga, the Tasman Front hits the North Island of New Zealand and splits into the East and West Auckland current (Figure 5) (Stevens et al. 2021; Papa et al. 2020). Around East Cape, the East Coast current originates from the East Auckland Current and flows southward around East Cape (Figure 5). Collectively, the recurrence of these genetic discontinuities across a diverse range of taxa suggests that Cape Reinga and the East Cape represent ecosystem‐wide biogeographic boundaries, which may further explain divergence in other marine taxa.

### 4.2 Genetic Connectivity Between Genetic Clusters

Our genomic analyses also provided insight into patterns of the dispersal and direction of gene flow across the genetic discontinuities. Direct evidence for migration across the genetic discontinuities was shown by dispersal events (i.e., individuals with contrasting genetic backgrounds compared to their sample location; Figure 2A). Dispersal events were observed in Cape Reinga, Gisborne, and Hawke's Bay, which were identified as mixing areas (Figure 5). Sample locations within the mixing areas exhibited intermediate levels of genetic differentiation (Figure 2B). Intermediate ancestry proportions indicate that the genetic variation in these sample locations originates from multiple ancestral populations (Figure 2C,D). Dispersal events across the genetic discontinuities were more frequently observed going from the NE cluster to the SW cluster, providing anecdotal evidence for asymmetrical dispersal (Figure 2C). Relative migration rates using neutral loci did not detect a difference in migration rates (Table S5). The ability to accurately estimate differences in migration using divmigrate may be limited due to low overall levels of genetic divergence. We hypothesize that strong ocean currents at both genetic discontinuities can possibly facilitate asymmetric gene flow (Figure 5), however, this remains largely speculative. Temporal variability in dispersal may also have limited the probability of detecting recent admixture events (Catalano et al. 2021; Peniston et al. 2024). Our sampling scheme did not allow us to test for the temporal variability of dispersal patterns. These observations raise the question of whether selection is influencing the observed patterns of gene flow among outlier loci. Notably, relative migration rates estimated using outlier loci showed 5% higher migration from the NE cluster to SW cluster (Table S5), suggesting that the gene flow of adaptive genetic variation is asymmetrical.

### 4.3 Identification of Local Adaptation

Signatures of local adaptation were observed in both genetic clusters despite high levels of population connectivity. Between genetic clusters, Manhattan plots of *F*_ST_ and *XP‐nSL* show 25 ROIs exhibiting both high levels of genetic differentiation (*F*_ST_: 0.0288–0.2582), compared to the genome‐wide average (*F*_ST_ = 0.0042), and substantial differences in the length of high‐frequency haplotypes (*XP‐nSL*: −10.56 to 8.85) (Figure 4; Table S6), providing strong support that these regions are under selection and involved in local adaptation. Positive selection observed in either genetic cluster is likely driven by differences in their oceanographic environments. New Zealand's heterogeneous seascape shows environmental gradients in ocean temperature, pH, salinity, and dissolved oxygen concentration that could facilitate adaptation to local environmental conditions (Figure S8). The identification of traits under selection is critical for inferring how genetic clusters adapt to their local environment. Most traits under selection in marine fish are polygenic (Bernatchez 2016), involving multiple genes that each contribute to an adaptive phenotype. For both genetic clusters, signatures of local adaptation were distributed across multiple ROIs (the NE cluster: 12; the SW cluster: 13), suggesting that traits under putative selection are polygenic. Nonetheless, GO enrichment analysis did not find any overrepresented GO terms among candidate genes. This does not imply that there is no connection between identified ROIs as the number of candidate genes is perhaps too small for us to confidently identify overrepresented GO terms. Furthermore, the annotation of NZ snapper may not yet be specific enough to allow a more gene set enrichment analysis. We therefore focused on a number of outlining genes and their functions to provide a tentative description of which traits may be under selection (Table S7). Candidate genes under putative selection in the NE cluster (positive *XP‐nSL*) showed overlap in high‐level gene functions that could not be associated with specific biological traits (e.g., hormone signaling and nervous system development). However, we identified four genes under putative selection in the SW cluster (negative *XP‐nSL*) across two ROIs on linkage groups 6 and 24 (pgm1, gyg1, mdh and ampkγ2). These candidate genes are involved in metabolic rate via glycogenesis (Table S7), which could potentially influence growth or other fitness‐related traits (Allendorf et al. 1983; Pereira et al. 2026; Townsend and Steinberg 2023). One notable observation is that in the most divergent ROI on linkage group 10, the candidate gene hk2 is suggested to be under selection in the NE cluster (*F*_ST_ = 0.2582). Hk2 is involved in glycolysis (Li et al. 2020) and is antagonistic to glycogenesis. Genes potentially related to growth are of interest, as this is a key phenotypic trait that is known to vary among snapper stocks within and between genetic clusters and is worth investigating (Parsons et al. 2014; Walsh 2003; Walsh and Davies 2004; Walsh et al. 2011, 2012).

**FIGURE 5:**
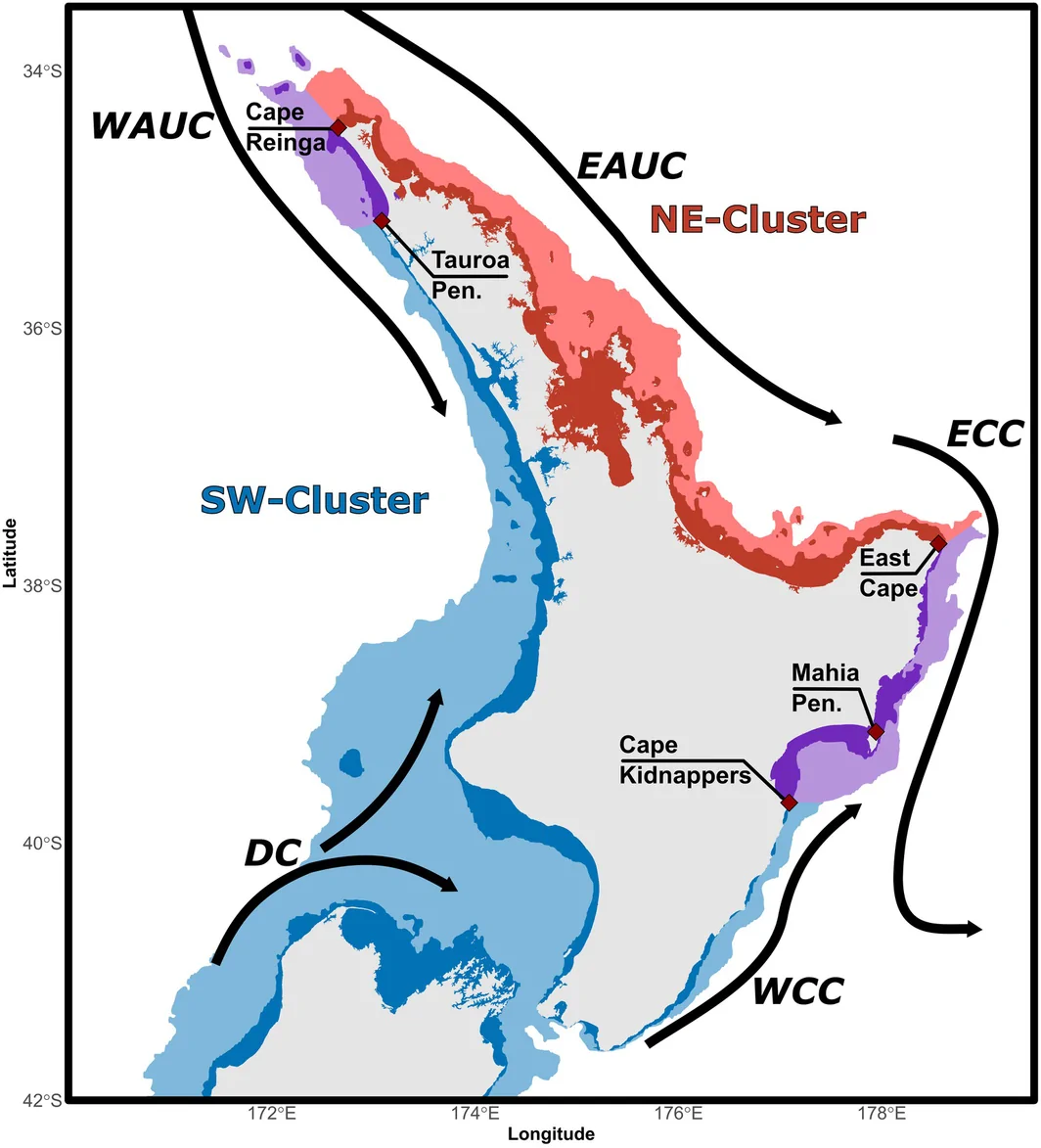
Spatial distribution of the East and West genetic clusters. Blue and red shaded areas show the 50 m bathymetry line for each of the genetic clusters, and light blue and light red shaded areas indicate the 200 m bathymetry line for each of the genetic clusters. The purple‐shaded area indicates where mixing of individuals from different clusters was observed and where genetic discontinuities occur over small spatial scales. Solid arrows indicate relevant ocean currents. EAUC, East Auckland Current; ECC, East Coast Current; DC, D'Urville Current; WAUC, West Auckland Current; WCC, Wairarapa Coastal Current.

The identification of local adaptation and the genes or traits involved in it is important for conservation management (Razgour et al. 2019). It is critical to understand how populations respond to their environment to assess their resilience and predict how they will respond to future changes as species are subjected to rapid environmental changes driven by anthropogenic threats. In New Zealand, ocean temperatures are increasing up to 34% faster than global averages (Ministry for the Environment and Stats NZ 2025; Pinkerton et al. 2024). Moreover, observed and ongoing changes in ocean circulation, ocean stratification, and primary production will likely affect how snapper interact with their environment. Our results show that snapper occur within two distinct genetic clusters and are potentially subjected to different environmental pressures, resulting in distinct genomic signatures of local adaptation (Figure 4). This provides an important baseline, which can help identify which snapper stocks will be most at risk under ongoing changes in global climate and develop proactive conservation strategies.

### 4.4 Monitoring Genetic Variation

The majority of genetic diversity was shared across all sample locations, with small but significant differences in heterozygosity between genetic clusters. Among neutral loci, 99% of genetic diversity was observed within individuals, and only 0.28% was observed between identified clusters (Table 1). This shows that most of the standing genetic variation is found across the entire distribution, minimizing the potential loss of genetic variation due to the local depletion of fish stocks (Razgour et al. 2019). Such levels of within‐species diversity are typical for marine species with high gene flow and large effective population sizes (Cano et al. 2008; Palumbi 2003; Ward 2000). Between genetic clusters, small but significant differences in levels of genetic variation were detected (Figure 3). Heterozygosity was higher in the NE cluster among high‐quality and neutral loci (Figure 3A,B). Small but significant differences are likely due to differences in population abundance, as stock biomass is higher in the NE cluster (Table S8), and thus able to maintain higher levels of genetic variation (Hoffmann et al. 2017). Interestingly, heterozygosity levels at outlier loci were higher in the SW cluster (Figure 3C). One hypothesis is that multiple adaptive alleles are being maintained in the SW cluster, which is distributed over a more heterogeneous seascape along a latitudinal axis (Figure S8).

Monitoring changes in genetic variation over time provides crucial information about the viability and resilience of snapper stocks (Chapman et al. 2009; Mimura et al. 2017). Loss of genetic diversity has been reported in the Tasman Bay area (Hauser et al. 2002), which had been heavily impacted by commercial fishing (Annala et al. 2000). However, subsequent analysis did not find significant differences between the Tasman Bay area and other stocks (Ashton 2013), suggesting that reductions could be due to temporal fluctuations or rapid recovery. Unfortunately, estimates of heterozygosity from microsatellite markers used in the previous studies are incompatible with our SNP data due to differences in mutational profiles and high mutation rates (Ellegren 2000), preventing us from testing for temporal changes in variation. Heterozygosity estimated from microsatellites ranged between 0.521 and 0.938, while estimates among neutral SNPs in this study ranged between 0.215 and 0.372 (Table S1). Future sampling and subsequent whole‐genome sequencing will be required to assess recent changes in genetic variation. Genomics studies have demonstrated that populations can maintain genetic variation even during short periods of intense exploitation (Therkildsen et al. 2010). Most notably, Pinsky et al. (2021) reported no loss of genetic variation in the heavily exploited North Atlantic cod. Like Atlantic cod, the period of exploitation for snapper was relatively short. These observations are encouraging, as they suggest that species (including snapper) can avoid long‐term loss of diversity from overexploitation. This study provides a baseline for future monitoring of snapper using SNPs, which better represent genetic variation within a species compared to highly mutagenic microsatellite loci. The development of monitoring programs for commercially and culturally important species, such as snapper, will contribute to their resilience to future climate change.

## 5 Conclusions

This study provided the first formal whole‐genome analysis of snapper in New Zealand, an economically and culturally important *taonga* species. We provide genome‐wide support for the presence of two distinct genetic clusters of snapper in New Zealand, with prominent genetic discontinuities around the Tauroa and Mahia Peninsulas, which are likely driven by ocean currents. The detection of dispersal events, low levels of genetic divergence, and substantial admixture indicates significant genetic connectivity between the two clusters and among demographically independent management stocks. Distinct signatures of selection across the entire genome imply that snapper have adapted to local environments despite low levels of overall population divergence and observations of ongoing gene flow. Tentative evidence for selection on polygenic traits associated with adaptation to local environments provides direction for future research projects to better understand how snapper respond to New Zealand's diverse and rapidly changing marine seascape. Finally, we advocate for continued monitoring of genetic variation using comparable genomic data in order to protect genetic diversity and safeguard resilience. Snapper are of major commercial, recreational, and cultural importance in New Zealand and are at risk due to significant changes in their marine environment. Taken together, this study provides new insights into how demographically independent stocks are genetically similar yet adapted to their local environments, which can help inform fisheries management policies that contribute to long‐term sustainable fishing practices amid rapid environmental change.

## Funding

This research was funded by the Royal Society of New Zealand Marsden Fund (Grant/Award Number: 16‐VUW‐040), Genomics Aotearoa, and European Union's Horizon 2020 4‐OCEANS Synergy grant (agreement no. 951649).

## Ethics Statement

No ethics approval was required for conducting this research. All biological material was obtained via commercial fisheries or competitive sports fishing events, and thus no fish were caught specifically for this research.

## Conflicts of Interest

The authors declare no conflicts of interest.

## Supporting information


**Figure S1:** Scree plot used to determine the number of informative principal components following Cattell's rule for the identification of outliers using pcadapt (*K* = 1).
**Figure S2:** Linkage decay plot. The *x*‐axis shows the distance between SNPs in base pairs, the *y*‐axis shows the average correlation between SNPs at the given genomic distance (black points), and the shaded area indicates the 95% quantile. The horizontal, red dashed line shows the commonly used threshold for the identification of linked sites (> 0.2). The vertical, black dashed line shows the sliding‐window size used for thinning our neutral dataset.
**Figure S3:** Coverage of each individual (A) before and (B) after normalization of bam files. Each point is an individual, and colors indicate sample locations.
**Figure S4:** PCA for three SNP datasets: High‐Quality, Neutral, and Outlier loci. PCAs show a consistent grouping of sample locations which cluster into two genetic clusters: the SW cluster and the NE cluster. A PCA based on a genetic relationship matrix using neutral loci shows genetic structure. Individuals were grouped by sample location. Ellipses indicate the spread of samples for each sample location, with lines that connect individual samples to the center of their respective ellipse.
**Figure S5:** Linearized genetic distance (FST) between sample locations plotted against geographic distance in km. Colors indicate whether comparisons were made between sample locations from the same cluster or between different clusters. The lines show the regression for each respective comparison. Top‐left shows the result from a correlation test for comparison, and the bottom right shows of the Mantel tests. Note that identity by distance is not detected within genetic clusters.
**Figure S6:** Cross‐validation scores from ADMIXTURE for selecting the most likely number of *K* populations. The lowest cross‐validation error indicates the most appropriate number of *K* is shown in red (*K* = 2).
**Figure S7:** Boxplots showing levels of heterozygosity for (A) high‐quality (B) neutral, (C) outlier loci, and (D) all sites per sample location. Sample locations that significantly differ are indicated by horizontal bars and their *p*‐value. Sample locations are ordered along the transect shown in Figure 1 of the main text: Karamea Bight (KB), Tasman Bay (TB), Marlborough Sounds (MB), Kapiti Coast (KC), West Coast (WC), Northland West (NW), Cape Reinga (CR), Northland East (NE), Hauraki Gulf (HG), Bay of Plenty (BP), East Cape (EC), Gisborne (GB), and Hawke's Bay (HB).
**Figure S8:** Plots showing the environmental gradient for the mean ocean temperature at max depth, pH, and the dissolved oxygen concentration at max depth. Figures show heterogeneous environmental gradients that pose selective pressures on snapper populations. Data have been obtained from the Bio‐Oracle database and plotted in R.

**Table S1:** Sample site information.
**Table S2:** Metadata for individuals.
**Table S3:** Pairwise‐distances between sample sites.
**Table S4:** Pairwise‐FST estimates between sample sites.
**Table S5:** Relative migration rates.
**Table S6:** Summary of regions of interest (ROI).
**Table S7:** Candidate genes and possible gene functions.
**Table S8:** Biomass estimates from Quota management areas and genetic clusters.

## Data Availability

Illumina short‐read data and associated metadata were deposited in the Genomics Aotearoa repository (https://repo.data.nesi.org.nz/), Repository ID: AGDR00033, in accordance with the principles of Māori Data Sovereignty (Te Aika et al. 2025). All scripts used in the analyses are publicly available on GitHub at https://github.com/tomoosting/EA2026_Snapper_Population_Genomics.
