# Supplementary material for: Fine‐Scale Population Structure, High Gene Flow and Local Adaptation in Australasian Snapper (Chrysophrys auratus) in New Zealand
Source: Evol Appl. 2026 Oct 22;19(10):e70327. doi: 10.1111/eva.70327 (PMC13689534; doi:10.1111/eva.70327)
Supplement: Supplementary file 1 [file EVA-19-e70327-s002.docx]

Figure S1: Scree plot used to determine the number of informative principal components following Cattell’s rule for the identification of outliers using pcadapt (K=1).

Figure S2: Linkage decay plot. X-axis shows distance between SNPs in base pairs, y-axis shows the average correlation between SNPs at the given genomics distance (black points), shaded area indicates the 95% quantile. Horizontal dashed red line shows the commonly used threshold for identification of linked sites (> 0.2). vertical black dashed line shows the sliding-window size used for thinning our neutral dataset.

Figure S3: Coverage of each individual A) before and B) after normalisation of bam files. Each point is an individual, and colours indicate sample locations.

Figure S4: PCA for three SNP datasets. High-Quality, Neutral, and Outlier loci. PCAs show a consistent grouping of sample locations which cluster into two genetic clusters, SW-cluster and NE-cluster. PCA based on a genetic relationship matrix using neutral loci showing genetic structure. Individuals were grouped by sample location. Ellipses indicate the spread of samples for each sample location, with lines that connect individual samples to the centre of their respective ellipse.

Figure S5: linearised genetic distance (F_ST_) between sample locations plotted against geographic distance in km. Colours indicate if comparisons were made between sample locations from the same cluster, or between different clusters. The lines shows the regression for each respective comparison. Top-left shows the result from a correlation test for comparison, bottom right shows of the Mantel tests. Note that identity by distance is not detected within genetic clusters.

Figure S6: Cross-validation scores from ADMIXTURE for selecting the most likely number of K populations. The lowest cross-validation error indicates the most appropriate number of K is shown in red (K=2).

Figure S7: Boxplots showing levels of heterozygosity for A) high-quality B) neutral, C) outlier loci and D) all sites per sample location. Sample locations that significantly differ are indicated by horizontal bars and p-value. Sample locations are ordered along the transect shown in figure 1 of main text: Karamea Bight (KB), Tasman Bay (TB), Marlborough Sounds (MB), Kapiti Coast (KC), West Coast (WC), Northland West (NW), Cape Reinga (CR), Northland East (NE), Hauraki Gulf (HG), Bay of Plenty (BP), East Cape (EC), Gisborne (GB) and Hawke’s Bay (HB).

Figure S8: Plots showing environmental gradient for mean ocean temperature at max depth, pH, and Dissolved oxygen concentration at max depth. Figures show heterogeneous environmental gradients that pose selective pressures on snapper populations. Data has been obtained from the Bio-Oracle database and plotted in R.
